# Comparison of Sea Snake (*Hydrophiidae*) Neurotoxin to Cobra (*Naja*) Neurotoxin 

**DOI:** 10.3390/toxins1020151

**Published:** 2009-12-03

**Authors:** Yumiko Komori, Masaya Nagamizu, Kei-ichi Uchiya, Toshiaki Nikai, Anthony T. Tu

**Affiliations:** 1 Department of Microbiology, Faculty of Pharmacy, Meijo University, Nagoya 468-8503, Japan; Email: kuchiya@ccmfs.meijo-u.ac.jp (K.U.); nikai@ccmfs.meijo-u.ac.jp (T.N.); 2 Department of Biochemistry and Molecular Biology, Colorado State University, Ft. Collins, CO 80523, USA

**Keywords:** *Praescutata viperina* toxin, Hydrophiinae toxin, sea snake toxin, amino acid sequence, computer modeling

## Abstract

Both sea snakes and cobras have venoms containing postsynaptic neurotoxins. Comparison of the primary structures indicates many similarities, especially the positions of the four disulfide bonds. However, detailed examination reveals differences in several amino acid residues. Amino acid sequences of sea snake neurotoxins were determined, and then compared to cobra neurotoxins by computer modeling. This allowed for easy comparison of the similarities and differences between the two types of postsynaptic neurotoxins. Comparison of computer models for the toxins of sea snakes and cobra will reveal the three dimensional difference of the toxins much clearer than the amino acid sequence alone.

## 1. Introduction

**Figure 1:**
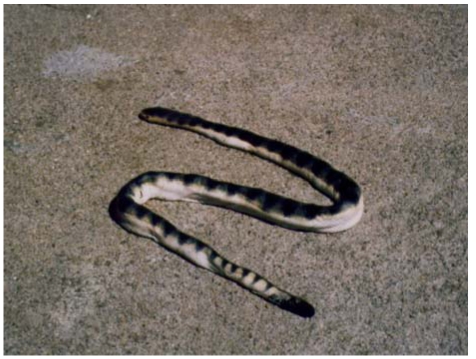
*Praescutata viperina* sea snake captured in 1989 in the Gulf of Thailand.

In tropical and subtropical regions, snakebites are a serious public health problem. The symptoms that follow snake envenomation are different depending on the species of snake. This is because the venoms are not entirely identical [1]. It has been known that venoms of Elapidae (cobras, kraits) land snakes are similar to that of Hydrophiidae (sea snakes) [2]. Venoms of both families contain potent postsynaptic neurotoxins. Close examination of the primary structures of neurotoxins obtained from Elapidae and Hyrophiidae showed considerable structural difference, although the backbone of the disulfide bonds is similar. In this investigation, we compared the neurotoxins of the two families by molecular model methods including the toxin obtained from *Prasecutata viperina* (Figure 1) that was newly isolated.

## 2. Materials and Methods

Specimens of the sea snake, *Praescutata viperina*, were captured in the Gulf of Thailand. The venom glands were removed and dried at room temperature. Crude venom was extracted from venom glands using distilled water and the insoluble tissue debris was removed by centrifugation (3,860 × g, 30 min, 4 °C), then the supernatant liquid was lyophilized for storage.

### 2.1. Isolation procedure

All of the purification procedures were performed at 4 °C. Crude venom (20 mg) was dissolved in 5 mL of 10 mM potassium phosphate buffer (pH 7.8) and applied to a CM52-cellulose column. The toxin was eluted with the same buffer at a flow rate of 13.5 mL per hour and it was monitored at 280 nm. Fractions of 3 mL were collected, and pooled fractions were tested for toxicity. The homogeneity of preparation was checked using SDS-polyacrylamide gel electrophoresis (SDS-PAGE) and reversed-phase HPLC. 

### 2.2. Toxicity test

The toxicity tests were done by injecting by 0.1 mL of toxin at various concentrations intravenously into ddY mice weighing 18 g each. At each of the five dosage levels, five mice were used. After 24 h, the number of mice that had died was observed. The toxicity was determined statistically using the method of Litchfield and Wilcoxon [3] and expressed as the lethal dosage 50%, the LD_50_ value (micrograms of toxin per gram of body weight of mouse). Experimental protocols concerning the use of laboratory animals were approved by the committee of Meijo University.

### 2.3. Biochemical characterization

The molecular mass of purified toxin was determined by MALDI/TOF-MS (matrix-assisted laser desorption/ionization time-of-flight mass spectrometry) with a Nihon Perseptive Biosystems/Voyager R.P., and by SDS-PAGE using the method of Weber and Osborn [4]. Isoelectric point was determined by the Pharmalyte concentration 3% (w/v) with a pH range of 3-10. Isoelectric foucusing was carried out using a constant potential of 200 V at 5 °C for 4 h.

The purified toxin (500 μg) was digested with endoproteinase Arg-C (5 μg) and 1% β-mercaptoethanol for 2 h at 37 °C in 1 mL of 100 mM Tris-HCl buffer (pH 7.8) containing 10 mM CaCl_2_. The purified toxin (500 μg) was digested with endoproteinase Lys-C (10 μg) for 24 h at 37 °C in 1 mL of 100 mM Tris-HCl buffer (pH 7.8) containing 1 mM EDTA. All digests were separated by reversed-phase HPLC. 

Various enzymatic activities and biological activity were determined by the following published procedures: Phospholipase A_2_[5], arylamidase [6], proteinase [7], arginine ester hydrolase [8], fibrinogenolytic [9], elastase [10], and hemorrhagic activities [11].

### 2.4. Sequence analysis

The amino-terminal sequence of native toxin and the enzymatically cleaved fragments of toxin were analyzed by an Applied Biosystems 491 protein sequencer. The phenylthiohydantoin (PTH) derivatives of amino acids were identified with an Applied Biosystems Model 610A PTH analyzer in accordance with the manufacturer’s instructions.

### 2.5. Homology analysis by computer modeling

MOE^TM^ (Molecular Operating Environment), a molecular simulation and modeling software purchased from Ryoka Systems Inc. was used for construction of protein models.

## 3. Results and Discussion

### 3.1. Isolation and purification

**Figure 2:**
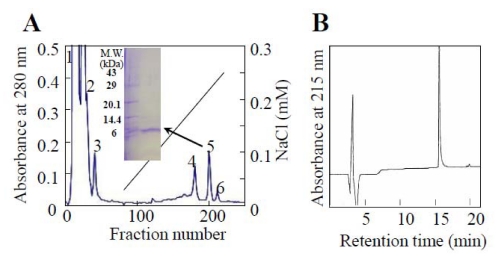
Elution profiles for the isolation of praescuta toxin from *Praescutata viperina* venom. (A) CM52-cellulose column chromatography. *Praescutata viperina* crude venom (20 mg) was applied to a column of CM52-cellulose (1.5 × 45 cm) equilibrated with 0.01 M potassium phosphate buffer (pH 7.8). The column was eluted with linear gradient from 0 to 0.3 M NaCl in a total volume of 600 mL of the same buffer. Fractions of 3.0 mL were collected at a flow rate of 13.5 mL/hr. SDS-PAGE of praescuta toxin under reduced condition. (B) Reversed-phase HPLC of praescuta toxin. The fraction 5 from CM52-cellulose column was applied to Develosil 300 ODS-HG-5 (4.6 × 250 mm) column equilibrated with solvent A (0.1% trifluoroacetic acid in H_2_O). Elution was achieved over 40 min with a linear gradient from 0 to 60% using solvent B (0.1% trifluoroacetic acid in acetonitrile) at a flow rate of 1.0 mL/min.

Praescuta toxin was isolated by a CM52-cellulose column chromatography (Figure 2) and lethal activity was found in fraction 5. Homogeneity of the toxin was established by two independent methods: SDS-PAGE (Figure 2A) and reversed-phase HPLC (Figure 2B). This purified toxin was named praescuta toxin and the yield of the praescuta toxin from 20 mg *Praescutata viperina* venom was found to be approximately 2.0 mg.

### 3.2. Toxicity

The LD_50_ of the praescuta toxin in mice was found to be 0.41 (0.31~0.55) μg/g by intravenous injection. Since the LD_50_ values of sea snake neurotoxins from *Pelamis platurus* and *Hydrophis ornatus* were reported to be 0.13 μg/g (intravenous injection) and 0.09 μg/g (intramuscular injection) respectively [12,13], the toxicity of *Praescutata viperina* toxin is relatively weak compared to sea snake neurotoxins. 

The effect of temperature on toxicity was also examined. A 50 μg of toxin preparation was heated for ten minutes at 70, 85 and 100 °C individually, and injected into mice (n = 3) intravenously. The results of injection are shown here:

70 °C: 3 mice died
85 °C: 1 mice died
100 °C: No mice died

It is well known that sea snake toxins are relatively stable during heat treatment because of their compact molecular structure with a relatively small size and a peptide backbone held together by four disulfide bonds [14]. The result shows the high thermal stability of the toxin. At 70 °C, all mice in the group died, which meant that the toxin retained full potency. Even at 85 °C, one out of three mice in the group died, indicating the toxin is still active even at high temperatures. The toxin was denatured in the test at 100 °C, as indicated by the survival of all three test animals.

### 3.3. Biochemical properties

The molecular mass of praescuta toxin determined by SDS-PAGE was found to be 7,400 Da, while the MALDI/TOF/MS method gave a molecular weight of 6,674.9 Da. The isoelectric point was determined by electrophoresis and was found to be higher than 10.0.

In order to ascertain that praescuta toxin is not an enzyme and does not show hemorrhagic and clotting activity, several assays were performed. The final preparation did not show phospholipase A_2_ activity, elastase activity using STANA (Suc-Ala-Ala-Ala-pNA) as the substrate, arginine ester hydrolase activity using TAME (tosyl-L-arginine methyl ester), arylamidase activity using L-leucine-β-naphtylamide as the substrate, proteinase activity using dimethylcaesin, fibrinogen, casein, and insulin B chains as substrates, collagenase activity using collagen Type IV as the substrate, and hemorrhagic activity. These results provide considerable evidence that this sea snake neurotoxin is a non-enzymatic type toxin. This conclusion is consistent with earlier findings that sea snake toxins bind to the acetylcholine receptor and are competitive inhibitors of acetylcholine [12,15].

### 3.4. Reduction of disulfide bonds

It is known that all sea snake toxins contain four disulfide bonds. Disulfide bonds are especially important for small proteins in order to hold the peptide backbone together. The reagent dithiothreitol (DTT) is known to cleave disulfides by reducing the bond. When 170 μg of praescuta toxin at a concentration of 17 μg /mL was incubated with 1% DTT at 37 °C for 30 min and then injected into three mice intraperitoneally, all of them survived. This indicates that the four disulfide bonds are essential for toxicity because the cleavage of these bonds with the reducing agent resulted in a loss of toxicity.

### 3.5. Direct amino acid sequence analysis and sequence analysis of digested fragments

**Figure 3:**
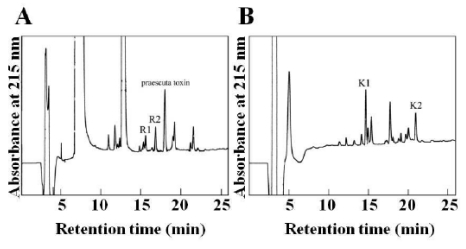
Fractionation of peptides obtained by digestion of praescuta toxin. The digestion of praescuta toxin was performed with endoproteinase Arg-C (A) and endoproteinase Lys-C (B). Elution conditions were the same as described in Figure 2B. The following abbreviations are used for the peptides: R1 and R2, endoproteinase Arg-C; K1 and K2, endoproteinase Lys-C.

Praescuta toxin was digested with endoproteinase Arg-C or endoproteinase Lys-C and different fragments were isolated by reversed-phase HPLC as shown in Figure 3A and Figure 3B.

The sequence of R1 and R2 fragments are:

Fragment R1: GWGXPQVKSGIKLE
Fragment R2: GTIIER

The sequence of K1 and K2 fragments are:

Fragment K1: LEXXHANEXNN
Fragment K2: TXRDHRGTIIERGWGXPQVK

**Figure 4:**
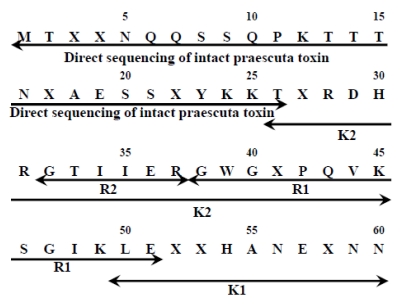
Amino acid sequence of praescuta toxin. Allows indicate residues determined by sequence analysis.

The purified toxin was also subjected to direct amino acid sequence analysis and the N-terminal sequence up to residue 26 was established (Figure 4). The “X“ denotes the unidentified cysteine residue by the PTH analyzer. 

By combining all the data obtained from the intact toxin and various fragments, the complete amino acid sequence of praescuta toxin was determined (Figure 4). From these results, praescuta toxin is composed of 60 amino acids and the molecular mass of the protein portion of praescuta toxin was calculated to be 6675.29 Da. The molecular mass of praescuta toxin based on the amino acid sequence was identical to the molecular mass (6674.9 Da) obtained by MALDI/TOF/MS.

### 3.6. Comparison of primary structure within Hydrophiinae toxins

**Figure 5:**
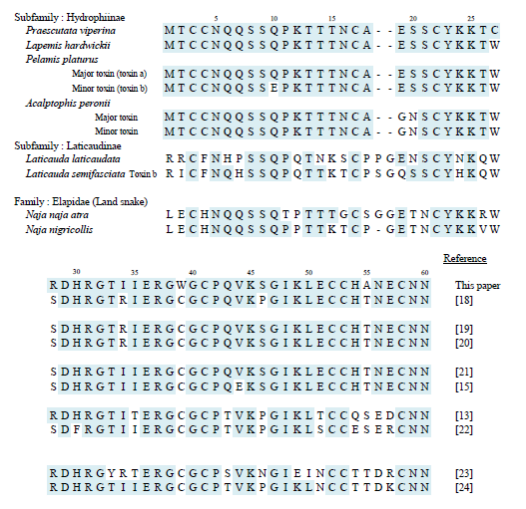
Comparison of amino acid sequence of praescuta toxin with several neurotoxins from sea snake and Elapidae snake.

The amino acid sequence of toxins isolated from *Lapemis, Pelamis and Acalptophis* venoms are extremely homologous and the positions of 9 cysteine residues are conserved (Figure 5), indicating that the three-dimensional structure of these toxins are similar. Praescuta toxin also possesses a similar primary structure with these toxins except for the position of Cys(27) and Trp(39). These substitutions of reactive amino acids might be the reason for the relatively weak toxicity of praescuta toxin.

The differences are few and are summarized here.

1. Position 10: Most toxins show Q (glutamine), with only *Pelamis platurus* minor toxin having E (glutamic acid).
2. Position 19: Either G (glycine) or E.
3. Position 34: Either I (isoleucine) or R (arginine).
4. Position 44: Most toxins have V (valine), and only one toxin shows E.
5. Position 46: Most are V; only *Lapemis hardwickii* shows P (proline).

### 3.7. Comparison of Hydrophiinae and Laticaudinae toxins

Only two toxins from the subfamily Laticaudinae are quoted for comparison (Figure 5). There are still many similarities, especially the disulfide bond backbone, but there are clearly more differences between the two subfamilies when analyzing the amino acid sequence of the neurotoxins than through an analysis of the sequences of toxins within the Hydrophiinae.

### 3.8. Comparison with land snake (Elapidae) toxins

Two cobra toxins are presented in Figure 5 for comparison. Elapidae toxins are quite different from all Hydrophiinae toxins. Comparison of the toxin sequences shown here reveals that their primary structure is related to the phylogenicity of the snakes: the more closely related, the more similar the amino acid sequence.

Recently Fry *et al*. [16] published a paper discussing the evolution of sea snakes extensively. In this paper, they mentioned that genus *Laticauda* is quite similar to many Elapidae land snakes. In our paper, our objective is more focused on chemical structure rather than evolution. We followed the classification of Smith [17], who mentioned that Hydrophiidae (sea snakes) contain two subfamilies of Hydrophiinae and Laticaudinae. 

### 3.9. Molecular modeling of praescuta toxin

**Figure 6:**
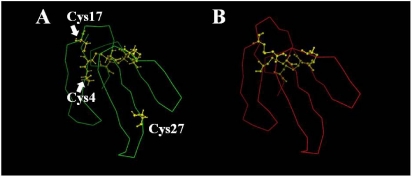
Molecular model of praescuta toxin. (A) Model structure of praescuta toxin presented with the position of nine cysteine residues. (B) Protein structure of α-neurotoxin from *Naja nigricollis* venom (PDB number 1IQ9; homology between praescuta toxin and α-neurotoxin was 68.3%).

**Figure 7:**
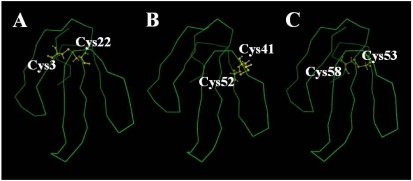
Molecular model of praescuta toxin with the speculated disulfide bonds. (A) Cys(3) and Cys(22), (B) Cys41 and Cys52, (C) Cys53 and Cys58. The position of disulfide bonds were speculated by comparison of praescuta toxin with α-neurotoxin from *Naja nigricollis* venom.

**Figure 8:**
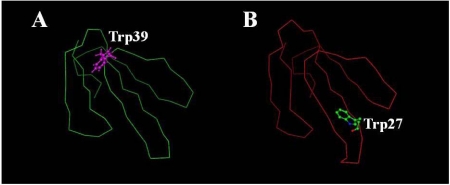
Presumption of the position of Trp(39) of praescuta toxin (A), and the position of Trp(27) exists on loop II of α-neurotoxin from *Naja nigricollis* venom.

Molecular modeling of praescuta toxin was performed using the information from samples of six neurotoxins which were selected from Protein Data Bank (PDB) as homologous proteins to this toxin. Figure 6A shows the protein model of praescuta toxin with the positions of nine cysteine residues. Comparing with α-neurotoxin from the venom of *Naja nigricollis* [24] (Figure 6B; PDB number 1IQ9), which had been chosen as the most homologous protein with 3-D sample information, Cys(27) of praescuta toxin exists at the tip of loopII. Since the positions of other eight cysteine residues of praescuta toxin [Cys(3), (4), (17), (22), (41), (52), (53) and (58)] are close each other, Cys(27) might exist alone without forming disulfide bonds. From the sequence data of α-neurotoxin indicated in Figure 5, the disulfide bonds of praescuta toxin are predicted to be formed between Cys(3) and (22), Cys(41) and (52), Cys(53) and (58) (Figure 7A-C). Cys(17) of praescuta toxin possibly bound with Cys(4), however, their positions on protein model are relatively apart (Figure 6A) and the formation of bonds between Cys(4) and (17) might cause an unstable structure. The substitution of Cys(27) and Trp(39) in praescuta toxin caused the lack of functionally important tryptophan residue to exist on loopII (Figure 8B), which participates in the stability of toxin-receptor complex. In addition, the existence of tryptophan residue at the position of 39 (Figure 8A) might induce conformational insecurity of praescuta toxin. Thus, these protein models elucidate the reasons of relatively weak toxicity of praescuta toxin clearly.
